# Propolis Efficacy: The Quest for Eco-Friendly Solvents

**DOI:** 10.3390/molecules27217531

**Published:** 2022-11-03

**Authors:** Ana Sofia Freitas, Ana Cunha, Pier Parpot, Susana M. Cardoso, Rui Oliveira, Cristina Almeida-Aguiar

**Affiliations:** 1 CITAB—Centre for the Research and Technology of Agro-Environmental and Biological Sciences, University of Minho, 4710-057 Braga, Portugal; 2 Department of Biology, School of Sciences, University of Minho, *Campus* de Gualtar, 4710-057 Braga, Portugal; 3 CBMA—Centre of Molecular and Environmental Biology, University of Minho, 4710-057 Braga, Portugal; 4 CQUM—Chemistry Center of the University of Minho, Department of Chemistry, University of Minho, *Campus* de Gualtar, 4710-057 Braga, Portugal; 5 CEB—Centre of Biological Engineering, University of Minho, *Campus* de Gualtar, 4710-057 Braga, Portugal; 6 LAQV-REQUIMTE, Department of Chemistry, University of Aveiro, 3810-193 Aveiro, Portugal

**Keywords:** propolis, propolis extraction, propolis extraction solvents, phenolic compounds, antibacterial activity, antioxidant potential

## Abstract

Propolis, a natural product made by bees with resins and balsams, is known for its complex chemical composition and remarkable bioactivities. In this study, propolis extraction was studied seeking extracts with strong bioactivities using less orthodox solvents, with some derived from apiary products. For that, a propolis sample collected from Gerês apiary in 2018 (G18) was extracted by maceration with six different solvents: absolute ethanol, ethanol/water (7:3), honey brandy, mead, propylene glycol and water. The solvent influence on the chemical composition and antioxidant and antimicrobial activities of the extracts was investigated. Antioxidant potential was assessed by the DPPH free-radical-scavenging assay and the antimicrobial activity by the agar dilution method. Chemical composition of the extracts was determined in vitro by three colorimetric assays: total ortho-diphenols, phenolics and flavonoids contents and the LC-MS technique. To our knowledge, this is the first time that solvents such as honey brandy and mead have been studied. Honey brandy showed considerable potential to extract propolis active compounds able to inhibit the growth of bacteria such as the methicillin-sensitive *Staphylococcus aureus* and *Propionibacterium acnes* (MIC values of 100 and 200 µg/mL, respectively) and the fungi *Candida albicans* and *Saccharomyces cerevisiae* (MIC = 500 µg/mL, for both). Mead extracts displayed high antioxidant capacity (EC_50_ = 1.63 ± 0.27 µg/mL) and great activity against resistant bacteria such as the methicillin-resistant *Staphylococcus aureus* and *Escherichia coli* (MIC = 750 µg/mL, for both). The production of such solvents made from beehive products further promotes a diversification of apiary products and the exploration of new applications using eco-friendly solutions.

## 1. Introduction

Propolis is a natural product composed by resinous and balsamic material collected by honeybees from different parts of plants and mixed with substances resulting from bees’ metabolism [1]. There is a long history of propolis use, which continues today in homemade remedies and personal products, as well as in over-the-counter products, mostly due to its vast list of preparations and uses. The demand for this beehive product is still increasing, along with the growing preference for natural products by the consumers. Propolis is commercialized in different parts of the world, being recognized as an important source of compounds with properties for several applications [2,3].

Most of the marketed propolis-based products require an extraction step, which allows the removal of inert material and the solubilization and preservation of the bioactive portion [4], enabling its introduction in pharmaceutical, food and cosmetic products [5]. In addition to the extraction method [6], the solvent used to extract the bioactive compounds is a determinant factor that influences the chemical composition of the obtained extracts [7]. Among the most used solvents—water, methanol, ethanol, chloroform, dichloromethane, ether and acetone—ethanol is considered the solvent of choice for obtaining propolis extracts with a high content of bioactive compounds [8]. However, ethanol has its drawbacks, such as a strong taste and limited applicability in certain industries, for instance, to treat ophthalmological diseases or in pediatric cases [9]. Different solvents extract distinct compounds [10], giving each extract particular properties and potentially specific applications. This work aims to highlight the importance of solvent selection, searching for new and more ecological solvents (e.g., honey brandy and mead, made from honey) that could promote a more sustainable beekeeping activity. To this end, a propolis raw sample collected in 2018 from Gerês (G18) was extracted with six different solvents: absolute ethanol, ethanol/water (7:3), honey brandy, mead, propylene glycol and water. The antioxidant potential and antimicrobial activity of the six extracts were evaluated, and the chemical composition was determined. Principal component analysis (PCA) was applied to evaluate possible associations between the chemical composition of propolis and bioactivities, with the aim of finding the main putative compounds responsible for such relevant activities.

## 2. Results and Discussion

### 2.1. Extraction Yield

Most studies on propolis have used ethanol to extract the bioactive compounds [4,11], and data on the chemical composition and bioactivities of non-ethanol propolis extracts are much more limited. In the present study, the chemical composition and antioxidant and antimicrobial activities of propolis extracts prepared with six different solvents*—*absolute ethanol, ethanol/water (7:3), honey brandy, mead, propylene glycol and water*—*were investigated. To our knowledge, this is the first time propolis was extracted with mead and honey brandy, two sweet-flavored fermented alcoholic drinks made from honey, water and yeast, with 40% and 15% of alcohol by volume (ABV), respectively. As presented in Table 1, the highest yields were obtained for absolute ethanol, ethanol/water (7:3) and propylene glycol extracts (>60%). Extraction with honey brandy resulted in a significantly lower yield (26.6%). However, the lowest yields were obtained for extraction with mead and water, the most polar solvent used (6.5 and 6.3%, respectively).

Although the extraction method was distinct from that used in the present work, ethanol was already described as the solvent able to obtain higher yield values, followed by propylene glycol and water [12]. However, a yield reported for water extraction was more than double the value obtained in this work, and for ethanol and propylene glycol extraction, it was around four-times lower [12]. Similar yield values for propolis water and ethanol extracts*—*1.81 and 51.03%, respectively*—*were found [13]. The same study also showed that the higher the alcohol content in the solvent, the higher the yield [13].

Yield comparisons between different works is always uncertain since extraction procedures are usually not performed under the exact same conditions. The extraction yield mainly varies with the technique and solvent used, but also depends on a combination of several other factors such as the ratio between the sample and the solvent, the fragmentation state of the sample and the agitation, temperature and time of extraction [14,15,16,17,18]. Although there is an increase in yield with increasing extraction time, this increase does not imply a significant increase in the phenolic content [14]. Propolis possess compounds with very different affinities to distinct solvents, for instance, compounds with more affinity to ethanol, such as the less polar active compounds, could not be easily extracted with polar solvents such as water, and vice versa. Ethanol is, however, a solvent with great affinity for a variety of compounds of different polarity, being consequently able to achieve higher yields [4,15].

### 2.2. Antimicrobial Activity

Antibacterial properties of the six G18 extracts*—*G18.EE, G18.EE_70_, G18.HBE, G18.ME, G18.PGE and G18.WE*—*were evaluated to assess the influence of the solvent on this activity (Table 2). Gram-positive bacteria, such as the bacteria of the genus Bacillus, were the most susceptible strains to all the extracts, which is a very common result reported by several authors, including our research group [19,20,21]. G18.EE, G18.EE_70_ and G18.PGE were the most active extracts, exhibiting the same MIC value against all the tested strains, followed by G18.HBE, which was slightly more active against the Gram-positive methicillin-sensitive Staphylococcus aureus (MSSA; MIC = 100 μg/mL) and Propionibacterium acnes (MIC = 200 μg/mL). G18.ME was, in general, one of the least active extracts, but the only one capable to inhibit the growth of the methicillin-resistant Staphylococcus aureus (MRSA; MIC = 750 μg/mL) and the Gram-negative bacterium tested, Escherichia coli (MIC = 750 μg/mL), proving to be particularly interesting to be used against these presumptive more resistant bacteria. The least active extract was the G18.WE, exhibiting the higher MIC values for all the tested strains (MIC ≥ 1000 μg/mL). The ethanol extracts, G18.EE and G18.EE_70_, as well as G18.HBE and G18.PGE, showed similar antibacterial activity when compared to ethanol extracts of propolis from Gerês from previous years [21].

The antifungal effect of the six G18 extracts—G18.EE, G18.EE_70_, G18.HBE, G18.ME, G18.PGE and G18.WE—was also evaluated to assess the influence of the solvent (Table 3). All extracts showed similar activity against both strains, with G18.HBE standing out from the others, being the most active against both *Saccharomyces cerevisiae* and *Candida albicans* (MIC = 500 μg/mL). This behavior is unusual for ethanol propolis extracts from Gerês [22], as the activity against these yeasts is normally weak, similar to the activity observed for the other extracts in this work. G18.WE was the least active extract against both yeasts (MIC > 2000 μg/mL).

Our results are in accordance with findings previously reported [9], showing that the propolis had EE_70_ higher activity against the Gram-positive bacteria over the Gram-negative. In addition, similar to our study, the extracts were particularly active against the spore-forming bacteria *Bacillus cereus* and no activity against all the tested strains was found for the propolis WE. Other studies also showed the inefficacy of water extracts of propolis as antimicrobial agents, showing weak or no activity at all against the tested strains, such as *Bacillus subtilis*, *B*. *cereus*, *S. aureus*, *Klebsiella pneumoniae*, *E. coli*, *Pseudomonas aeruginosa* and *C. albicans* [9,23]. On the other hand, ethanol and propylene glycol demonstrated potential as extraction solvents to obtain propolis extracts with great antimicrobial activity, especially against *S. aureus* and *B. subtilis* [23]. Two different propolis extracts*—*ethanol and water*—*were tested, and MIC values in the same range as ours were obtained against *S. aureus* (250 and 1000 μg/mL, for propolis EE and WE, respectively) and E. coli (1000 and > 1000 μg/mL for the propolis EE and WE, respectively) [8].

### 2.3. Antioxidant Activity

DPPH free-radical-scavenging activity was determined for the six G18 extracts*—*G18.EE, G18.EE_70_, G18.HBE, G18.ME, G18.PGE and G18.WE*—*and the respective EC_50_ values were calculated (Table 4). G18.EE and G18.EE_70_ showed similar and lower anti-radical activity (10.78 ± 0.43 and 9.91 ± 1.07 µg/mL, respectively) than G18.PGE (8.17 ± 0.14 µg/mL). G18.HBE and G18.WE exhibited similar values of EC_50_ (4.90 ± 0.10 and 4.54 ± 0.23 µg/mL, respectively) and a high antioxidant potential, but G18.ME displayed the highest antioxidant potential with a particularly low EC_50_ value of 1.63 ± 0.27 µg/mL. Contrary to what happen for the antibacterial activity, where the G18.WE and G18.ME were the weaker extracts against all tested bacterial strains, these extracts exhibited the higher capability to scavenge DPPH free radicals, suggesting that extracts with lower antibacterial activity usually have higher antioxidant potential.

Other authors have evaluated the DPPH free-radical-scavenging activity of propolis ethanol and water extracts [9,13,24,25,26,27], reporting their high antioxidant potential. However, the literature does not point to a predominance of EE or WE for higher antioxidant activity, with studies finding higher scavenging ability of WE when compared to the EE of the same propolis sample, similar to this work, whereas others have found EE with greater antioxidant capacity instead (IC_50_ of 695 and 13*,*798 µg/mL, for the EE and WE respectively) [9,13,27]. However, the value obtained for the G18.EE fits into the scavenging activity exhibited by the EE from Gerês from previous years, supporting the constancy exhibited by these particular propolis samples [21].

### 2.4. Chemical Characterization

#### 2.4.1. In Vitro Evaluation of Total Ortho-Diphenols (TOC), Phenolics (TPC) and Flavonoids (TFC) Contents

Table 5 summarizes the total ortho-diphenols (TOC), phenolics (TPC) and flavonoids (TFC) contents of the G18 extracts. TOC ranged from 263.05 ± 15.19 to 1067.79 ± 37.24 mg GAE/g extract. The lowest value achieved for absolute ethanol and ethanol/water (7:3) extracts, while the highest TOC was achieved by the G18.WE and G18.ME. TPC values ranged from 207.49 ± 8.55 to 1267.57 ± 5.27 mg GAE/g extract, with G18.PGE exhibiting the lower value, while the highest TPC value was displayed, again, by G18.WE and G18.ME. TFC values ranged from 7.40 ± 0.14 mg QE/g extract for G18.WE to 50.97 ± 0.26 mg QE/g extract for G18.HBE. TOC correlated directly to the antioxidant potential as the extracts with higher TOC exhibited higher ability to scavenge DPPH free radicals. On the other hand, TFC seems to be linked, although not directly correlated, to the antibacterial activity, as extracts with higher TFC generally showed stronger antibacterial activity against the different bacterial strains tested.

G18.EE showed TPC and TFC values similar to the ones reported for G.EEs from previous years [22]. Our results showed slight differences from what was previously described [15,23,28,29], indicating that ethanol extraction leads to higher TPC and TFC values when compared to water extraction. Although both G18.EE and G18.EE_70_ showed higher TFC values than G18.WE, the latter showed a value of TPC around five-times higher than the ethanol extracts. The values of TPC and TFC found in the literature for EE are in the range of the values obtained in the present study. The same is not true regarding WE since different authors have reported distinct values for their extracts. For instance, some authors have reported TPC values for propolis EE in the range of the values found in our study (152.03 and 231.92 mg GAE/g extract, respectively) but lower values for the WE (6.68 and 61.92 mg GAE/g extract, respectively) [13,28]. The same tendency has been described by other authors who reported TPC values of 321 and 210 mg GAE/g extract for the propolis EE and WE, respectively [27], and TPC values of high range for both propolis EE (1207.9 µg/mL) and WE (20791.3 µg/mL) [29]. Nevertheless, the TPC values found in the literature for WE are generally much lower than this last value and the values obtained in the present work. However, and similar to our work, similar TPC values for both ethanol and propylene glycol extracts have been described and, when compared to the water extract of the same propolis sample, both ethanol and glycol propolis extracts have shown higher TFC [12,23,28].

#### 2.4.2. UHPLC-DAD-ESI/MS^n^ Analysis

After liquid chromatography-mass spectrometry (LC-MS) analysis of the different extracts of propolis from Gerês, 51 compounds were detected (see Supplementary Material Figure S1 and Table S1). Regardless the differences found in the total ortho-diphenols, phenolics and flavonoids contents, most of the different solvent extracts showed similar phenolic constituents, particularly the extracts with higher alcohol content—G18.EE, G18.EE_70_ and G18.HBE—and G18.PGE. Non-ethanol solvents of glycolic nature, which is the case of propylene glycol and polyethylene glycol, have been shown to be more efficient in extracting the active portion from propolis when compared to ethanol [9]. Among all compounds, only two, gallic acid and caffeic acid, were detected in all the extracts. Gallic acid, caffeic acid, *p*-coumaric acid, ferulic acid, 3,4-dimethyl-caffeic acid (DMCA), quercetin-3-methyl ether, apigenin, chrysin, caffeic acid isoprenyl ester (CAIE), acacetin and pinocembrin were the main compounds found, among many others also observed in the chemical profiles described for European propolis [21], typical for poplar propolis ethanol extracts [11].

Both G18.ME and G18.WE exhibited distinct chemical profiles. The G18.WE was mainly composed by tannins and coumaric acid derivatives, whereas the few phenolic compounds detected in G18.ME consisted of phenolic acids and hydroxycinnamic acids, namely gallic acid, caffeic acid, ellagic acid, *p*-coumaric acid, ferulic acid, 3,4-dimethyl-caffeic acid (DMCA) and *p*-coumaric acid methyl ester. As this extract is characterized by a potent antioxidant capacity (EC_50_ = 1.63 ± 0.27 µg/mL), it is tempting to associate the presence of these phenolic acids to this property, which is in line with what has already been described in the literature [11]. Still, the activity against the MRSA and *E*. *coli* can also be related to the presence of the same compounds. Thus, it would be interesting to test several combinations of the seven compounds to find the major putative compounds responsible for the activity against these two resistant bacteria.

### 2.5. Multivariate Analysis

**Figure 1:**
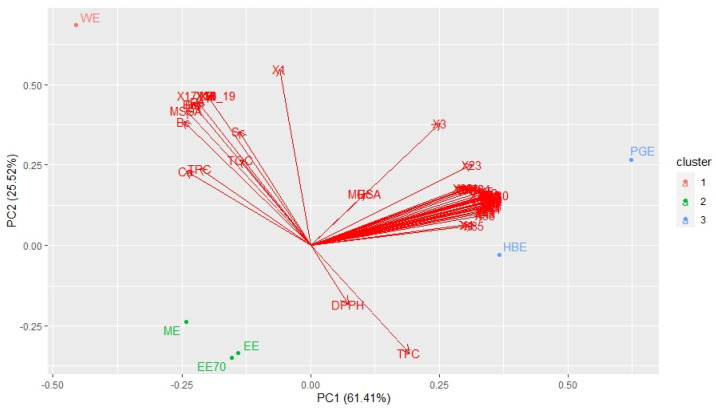
PC2 vs. PC1 biplot graph for the propolis samples and selected biochemical parameters. **EE**: ethanol extract; **EE_70_**: ethanol/water (7:3) extract; **HBE**: honey brandy extract; **PGE**: propylene glycol extract; **ME**: mead extract; **WE**: water extract; **DPPH**: DPPH free-radical-scavenging activity expressed as a mean of EC_50_ values (μg/mL); **TFC**: total flavonoids content; **TPC**: total phenolics content; **TOC**: total ortho-diphenols content; Microorganisms—**Bs**: *Bacillus subtilis*; **Bc**: *Bacillus cereus*; **Bm**: *Bacillus megaterium*; **MSSA**: methicillin-sensitive *Staphylococcus aureus*; **MRSA**: methicillin-resistant *Staphylococcus aureus*; **Pa**: *Propionibacterium acnes;* **Ec**: *Escherichia coli*; **Sc**: *Saccharomyces cerevisiae*; **Ca**: *Candida albicans*—distributed according to the MIC values; **x1—x51**: compounds detected by UHPLC-DAD-ESI/MS^n^ analysis (see Supplementary Material Table S1).

Principal Component Analysis (PCA) was carried out to find possible correlations between the presence of particular groups of compounds and the antioxidant as well as antimicrobial activities. The PC1 and PC2 represented 61.41 and 25.52% of the total variance, respectively. The biplot graph for the first two PCs is presented in Figure 1. Considering the similarities of the samples and the variables related to the chemical composition of propolis extracts and their antioxidant and antimicrobial activities, the first ones could be divided into three groups (Figure 1). The first group is composed by the absolute ethanol (EE), ethanol/water (7:3) EE_70_) and the mead (ME) extracts; the second group by the honey brandy (HBE) and propylene glycol (PGE) extracts; and the third group by the water extract (WE) alone, as the most differentiated extract.

**Figure 2:**
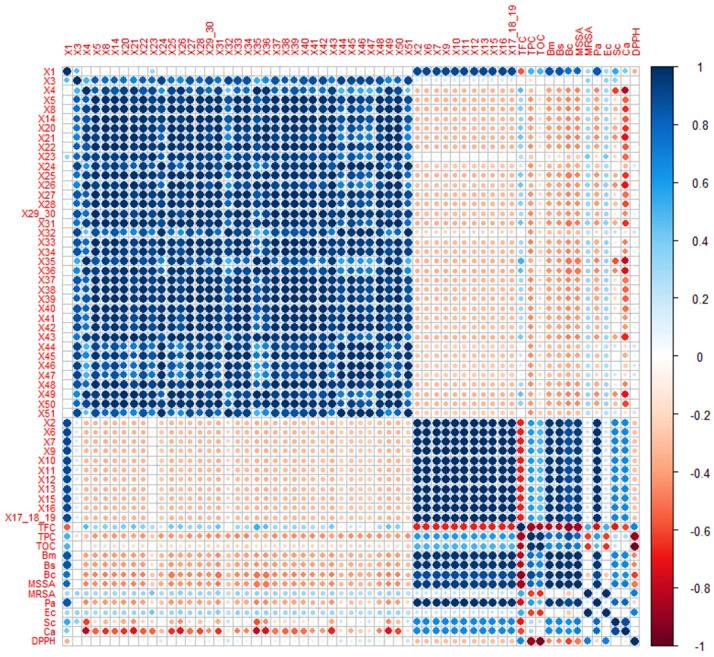
Correlation plot of the chemical composition and bioactivities of the propolis extracts. Dark blue (positive correlation) and dark red (negative correlation) show the strength and direction of the correlations between the variables. **DPPH**: DPPH free-radical-scavenging activity expressed as a mean of EC_50_ values (μg/mL); **TFC**: total flavonoids content; **TPC**: total phenolics content; **TOC**: total ortho-diphenols content; Microorganisms—**Bs**: *Bacillus subtilis*; **Bc**: *Bacillus cereus*; **Bm**: *Bacillus megaterium*; **MSSA**: methicillin-sensitive *Staphylococcus aureus*; **MRSA**: methicillin-resistant *Staphylococcus aureus*; **Pa**: *Propionibacterium acnes;* **Ec**: *Escherichia coli*; **Sc**: *Saccharomyces cerevisiae*; **Ca**: *Candida albicans*—distributed according to the MIC values; **x1–x51**: compounds detected by UHPLC-DAD-ESI/MS^n^ analysis (see Supplementary Material Table S1).

As depicted in Figure 2, it is possible to confirm that TOC and TPC are both inversely proportional to DPPH, indicating a correlation between these first ones and antioxidant potential. To our knowledge, this is a new finding since no information was found in the literature concerning TOC analysis of propolis samples and considering that the antioxidant activity is usually positively correlated with the total flavonoids and phenolics contents [26]. The TFC is strongly correlated with the antibacterial activity, particularly related to the Gram-positive bacteria—*B*. *subtilis*, *B*. *cereus*, *B*. *megaterium*, MSSA and *P*. *acnes*—and the antifungal—*S*. *cerevisiae* and *C*. *albicans*. On the other hand, the angles between the other two variables, TPC and TOC, and MRSA and the gram-negative bacterium *E*. *coli*, are approximately 90°, indicating correlation coefficients close to 0 (Figure 1). These results show that TPC and TOC are not correlated with MRSA and *E*. *coli*, and the response for this last one could not be predicted from propolis TPC and TOC.

Both antioxidant and antimicrobial activities are universally exhibited by propolis, and the same happens with some of the compounds that are part of this complex mixture, such as gallic acid, ellagic acid, ferulic acid, isorhamnetin, kaempferol, caffeic acid isoprenyl ester (CAIE), caffeic acid phenethyl ester (CAPE) and tannins [13,30,31,32,33,34,35,36,37,38,39,40,41,42,43,44,45]. The presence of specific phenolic compounds, such as gallic acid (compound **x1**), present in all the extracts but in different quantities, and the HHDP-hexoside (**x2**), digalloyl hexoside (**x6**), gallotannin (**x7**), di(HHDP-galloylglucose)-pentose (**x9**), tannins (**x10–12, x15**), caffeic acid derivatives (**x13**) and coumaric acid derivatives (**x16–19**), only present in G18.WE, although not individually correlated, could, acting synergistically, be responsible for the strong correlation between the G18.WE and the antioxidant potential (DPPH; Figure 1 and Figure 2). In contrast, the antimicrobial activity, more specifically against the fungi *S*. *cerevisiae* and *C*. *albicans*, and the bacteria of the genus *Bacillus* and the *S. aureus* and *P*. *acnes*, seems to be linked to the presence of other group of compounds, mostly flavonoids, such as the ellagic acid (**x4**), ferulic acid (**x8**), pinobanksin-5-methyl-ether (**x20**), quercetin-3-methyl ether (**x21**), kaempferol (**x25**), isorhamnetin (**x26**), acacetin (**x31**), kaempferide (**x35**), kaempferol-methoxy-methyl ether (**x36**), 3-hydroxy-5-methoxy flavanone (**x43**) and caffeic acid derivatives (**x49**).

## 3. Materials and Methods

### 3.1. Propolis Sample and Extracts Preparation

The propolis used in this work was harvested in 2018 from an apiary located in Gerês, in the north of Portugal and near the Cávado River (41°45′41.62′’ N; 7°58′03.34′’ W), a protected area of the Peneda-Gerês National Park [21]. Beekeeping is performed under a certified biological mode and propolis is produced and harvested from grids by a standardized method, being of high quality and value.

The sample, named G18, was carefully cleaned of debris and finely fragmented before being extracted by maceration as previously described [21] but using distinct solvents—absolute ethanol, ethanol/water (7:3), honey brandy, mead, propylene glycol and water—to achieve different extracts—ethanol extract (EE), ethanol/water (7:3) extract (EE_70_), honey brandy extract (HBE), mead extract (ME), propylene glycol extract (PGE) and water extract (WE). Homemade honey brandy and mead were obtained using honey from the same region. A volume of 60 mL of each solvent was added to approximately 9 g of propolis and the mixtures were placed under orbital agitation (100 rpm), at 24 °C, in the dark, for 24 h. The mixtures were then filtered under a vacuum, and the filtrates were reserved at 4 °C. The solid residues were extracted again with 40 mL of the respective previously used solvent. The resulting filtrates were pooled, and the following extracts were obtained: G18.EE, G18.EE_70_, G18.HBE, G18.ME, G18.PGE and G18.WE. At the end of the process, three 500 µL aliquots of each extract were dried under nitrogen flow for yield analysis.

### 3.2. In Vitro Characterization of the Chemical Composition of Extracts of Propolis from Gerês Harvested in 2018

The six propolis extracts were characterized regarding their phenolic composition using spectrometric and chromatographic methodologies.

#### 3.2.1. Total Ortho-Diphenols Content (TOC)

The content in ortho-diphenol compounds was determined using an adaptation of a colorimetric assay [46]. In brief, an identical volume of different concentrations (from 25 to 300 µg/mL) of each of the six G18 extracts were mixed with 5% (*w*/*v*) sodium molybdate, in ethanol/water 1:1 (*v*/*v*), (following a 4:1 proportion in the reaction mixture), followed by 15 min incubation at room temperature in the dark. Control and blank were prepared with similar mixtures but with each solvent replacing the extract or sodium molybdate, respectively. Absorbance of the reaction was measured at 370 nm, and results were obtained from linear regression using gallic acid as the standard (calibration curve with concentrations ranging from 40 to 180 µg/mL). TOC is expressed in milligrams of gallic acid equivalents (GAE) per gram of propolis extract (mg GAE/g extract).

#### 3.2.2. Total Phenolics Content (TPC)

The content in phenolic compounds was determined using an adaptation of the Folin-Ciocalteu colorimetric assay [31]. Briefly, different concentrations (from 1 to 300 µg/mL) of each of the six G18 extracts were mixed with 10% (*v*/*v*) Folin-Ciocalteu reagent and 7.5% (*w*/*v*) Na_2_CO_3_ (following a 5:5:4 proportion in the reaction mixture). The control and the blank were prepared with similar mixtures but with each solvent replacing the extract or reagents, respectively. Absorbance was measured at 760 nm after 1 h incubation at room temperature in the dark, and results were obtained from linear regression using gallic acid as the standard (calibration curve with concentrations ranging from 1 to 50 µg/mL). TPC is expressed in milligrams of gallic acid equivalents (GAE) per gram of propolis extract (mg GAE/g extract).

#### 3.2.3. Total Flavonoids Content (TFC)

The content in flavonoid compounds was determined using an adaptation of the colorimetric assay previously described [31]. In brief, different concentrations (from 200 to 2200 µg/mL) of each of the six G18 extracts were mixed with 2% (*w*/*v*) AlCl_3_ (following a 1:1 proportion in the reaction mixture). Control and blank were prepared with similar mixtures but with each solvent replacing the extract or AlCl_3_, respectively. The absorbance of the reaction was measured at 420 nm after 1 h incubation at room temperature in the dark, and results were obtained from linear regression using quercetin as the standard (calibration curve with concentrations ranging from 5 to 200 µg/mL). TFC is expressed in milligrams of quercetin equivalents (QE) per gram of propolis extract (mg QE/g extract).

### 3.3. Analysis of G18 Extracts Phenolic Compounds by UHPLC-DAD-ESI/MS^n^

The phenolic profile of the extracts was elucidated by UHPLC-DAD-ESI/MS^n^ analysis following the method previously used [21]. Analysis was performed on an Ultimate 3000 (Dionex Co., Rockville, MD, USA) apparatus equipped with an ultimate 3000 Diode Array Detector (Dionex Co., Rockville, MD, USA) coupled to a mass spectrometer LTQ XL Linear Ion Trap 2D. The chromatographic system comprised a quaternary pump, an autosampler, a photodiode-array detector and an automatic thermostatic column compartment. The mass spectrometer was a Thermo LTQ XL (Thermo Scientific, Waltham, MA, USA) ion trap MS equipped with an ESI source. Compounds were identified by comparison of the ESI-MS/MS with the data from MS/MS published in the literature [21,47,48,49,50,51,52].

### 3.4. In Vitro Evaluation of the Antioxidant Potential of Extracts of Propolis from Gerês Harvested in 2018

The DPPH^•^ (2,2-diphenyl-1-picryl-hydrazyl) method was used for the in vitro evaluation of the antioxidant potential of the propolis extracts as previously described [21]. In brief, different concentrations (from 1 to 50 µg/mL) of each of the six G18 extracts were mixed with 0.004% (*w*/*v*) DPPH^•^ in a proportion of 1:2. The control and the blank were prepared with similar mixtures but with each solvent replacing the extract or DPPH^•^, respectively. Absorbance was measured at 517 nm after 20 min incubation at room temperature in the dark, and the results are expressed in EC_50_ values (µg/mL; concentration that reduce the free radical in 50%) from linear regression of the percentage decrease in absorbance with respect to the control values.

### 3.5. Antimicrobial Properties of G18 Extracts—Determination of Minimum Inhibitory Concentrations

Minimum inhibitory concentrations (MIC) of the six G18 extracts were determined using the agar dilution method [53] and the following panel of indicator strains: the Gram-negative bacterium *Escherichia coli* CECT 423, six Gram-positive bacteria—*Bacillus subtilis* 48886, *Bacillus cereus* ATCC 7064, *Bacillus megaterium* 932, (methicillin-snsitive) *Staphylococcus aureus* ATCC 6538 (MSSA), (methicillin-resistant) *Staphylococcus aureus* M746665 (MRSA) and *Propionibacterium acnes* H60803—as well as two yeast strains: *Saccharomyces cerevisiae* BY4741, from Euroscarf (http://euroscarf.de/index.php?name=Description; accessed on 22 July 2021), and *Candida albicans* 53B, from the microbial collection of the Department of Biology of the University of Minho.

Bacteria and yeast were cultured, respectively, in LB (Luria-Bertani—0.5% (*w*/*v*) yeast extract, 1% (*w*/*v*) tryptone, 1% (*w*/*v*) NaCl) and YPD (1% (*w*/*v*) yeast extract, 2% (*w*/*v*) peptone, 2% (*w*/*v*) glucose) broths or in solid media (LBA and YPDA—by adding 2% (*w*/*v*) agar to the previous recipes of LB and YPD, respectively). Growth was performed at 200 rpm—at 37 °C for bacteria and 30 °C for yeast—and monitored by optical density at 600 nm (OD_600_). To prepare the cells for the experiments, overnight microbial cultures were diluted with the appropriated fresh medium to an OD_600_ = 0.1 and incubated under the same conditions until the OD_600_ reached 0.4–0.6 (mid-exponential phase).

Drops of 5 µL of exponentially growing microbial cultures were transferred onto LBA and YPDA plates (for bacteria and yeast, respectively), supplemented with different concentrations of each of the six G18 extracts (0, 10, 50, 100, 200, 500, 750, 1000, 1250, 1500, 1750 or 2000 µg/mL). Plates containing identical volumes of each solvent were used as controls. Plates were incubated at 37 °C for 24 h for bacteria and at 30 °C for 48 h in the case of yeast. MIC values were determined upon observation of the presence/absence of growth.

### 3.6. Principal Component Analysis

Principal Component Analysis (PCA) was performed for multivariate data analysis using R Studio version 1.4.1106 (Copyright 2009-2021 R Studio, PBC), R version 4.0.5 (Copyright (C) 2021 The R Foundation for Statistical Computing). The data matrix was composed by 6 samples (propolis extracts) and 64 variables, including the compounds detected (51 compounds) by UHPLC-DAD-ESI/MS^n^, the total phenolics (TPC), flavonoids (TFC) and ortho-diphenols contents (TOC) and the antioxidant potential (DPPH), as well as the antimicrobial activity (MIC values). The correlation between variables and the relationship between samples and biochemical parameters were determined using scores, loadings and biplot graphs.

### 3.7. Statistical Analysis

Antimicrobial and antioxidant experiments were performed in triplicate, repeated at least three times independently, and results were expressed as mean ± standard deviation (SD). A one-way ANOVA followed by Tukey’s test for multiple comparisons were used to assess treatment effect. Differences considered statistically significant (*p* < 0.05) were noted with different letters.

## 4. Conclusions

In general, ethanol extracts of propolis from Gerês appear to be more suitable for antimicrobial applications, which correlates with their high content in flavonoids, while the mead extract seems to be a more promising choice concerning the antioxidant potential, which correlates with its higher content in phenolic acids. Solvents such as honey brandy and mead must be further explored. Beyond the strong antioxidant potential of the corresponding propolis extracts obtained with such solvents, they possess considerable infections-fighting potential, namely fungal, similar to the infections caused by *C*. *albicans*, and bacterial, particularly against some resistant bacteria such as MRSA and *E*. *coli*. As different solvents can extract not only distinct amounts of the same compound but also diverse groups of compounds, this study can contribute to a more conscious choice of the appropriate solvent in order to extract a propolis active portion according to the desired applicability. The increasing health-conscious awareness of consumers regarding the potential toxicity of the chemicals used in their daily life has led to the quest for more natural and sustainable alternatives. Solvents such honey brandy and mead, made from natural sources such as bee products encompasses bioeconomy, are also an additional source of income for the beekeeping sector as alternative uses for surplus honey production and/or reuse of products that may not have commercial value to be marketed, as these solvents can be marketed an eco-friendly alternative to the current solvents in use.

## Figures and Tables

**Table 1:** Yields (%) resulting from the extraction of G18 with different solvents—absolute ethanol (G18.EE), ethanol/water (7:3) (G18.EE_70_), honey brandy (G18.HBE), mead (G18.ME), propylene glycol (G18.PGE) and water (G18.WE)—expressed as a mean and respective standard deviation (SD). Statistical analysis was performed by one-way ANOVA followed by Tukey’s test for multiple comparisons. Mean values with the same letters (a, b or c) are not statistically different.

Propolis Extracts	G18.EE	G18.EE_70_	G18.HBE	G18.ME	G18.PGE	G18.WE
Yield (%)	61.6 ± 1.2 ^a^	68.3 ± 0.7 ^a^	26.6 ± 2.8 ^b^	6.5 ± 1.0 ^c^	64.0 ± 5.2 ^a^	6.3 ± 0.7 ^c^

**Table 2:** MIC values (µg/mL) obtained against the panel of susceptibility indicator bacteria. Mid-exponential phase microbial cultures were transferred to plates supplemented with increasing concentrations of each of the six extracts—G18.EE, G18.EE_70_, G18.HBE, G18.ME, G18.PGE and G18.WE. Plates were observed for the presence/absence of growth after 24 h incubation at 37 °C, and the lowest concentrations for which no growth was detected were registered as the MIC values.

Bacteria	G18.EE	G18.EE_70_	G18.HBE	G18.ME	G18.PGE	G18.WE
	MIC (µg/mL)					
*Bacillus megaterium*	50	50	50	500	50	2000
*Bacillus subtilis*	50	50	50	500	50	2000
*Bacillus cereus*	50	50	50	500	50	1000
MSSA	200	200	100	750	200	2000
MRSA	>2000	>2000	>2000	750	>2000	>2000
*Propionibacterium acnes*	500	500	200	500	500	2000
*Escherichia coli*	>2000	>2000	>2000	750	>2000	>2000

MSSA—methicillin-snsitive *Staphylococcus aureus*; MRSA—methicillin-resistant *Staphylococcus aureus*; EE—ethanol extract; EE_70_—ethanol/water (7:3) extract; HBE—honey brandy extract; ME—mead extract; PGE—propylene glycol extract; WE—water extract.

**Table 3:** MIC values (µg/mL) obtained against the two-susceptibility indicator yeasts. Mid-exponential phase microbial cultures were transferred to plates supplemented with increasing concentrations of each of the six extracts—G18.EE, G18.EE_70_, G18.HBE, G18.ME, G18.PGE and G18.WE. Plates were observed for the presence/absence of growth after 48 h incubation at 30 °C, and the lowest concentrations for which no growth was detected were registered as the MIC values.

Yeast	G18.EE	G18.EE_70_	G18.HBE	G18.ME	G18.PGE	G18.WE
	MIC (µg/mL)					
*Saccharomyces cerevisiae*	1500	1500	500	1500	2000	>2000
*Candida albicans*	2000	1500	500	1500	1500	>2000

EE—ethanol; EE_70_—ethanol/water (7:3) extract; HBE—honey brandy extract; ME—mead extract; PGE—propylene glycol extract; WE—water extract.

**Table 4:** Antioxidant potential of G18.EE, G18.EE_70_, G18.HBE, G18.ME, G18.PGE and G18.WE, measured by the in vitro DPPH free-radical-scavenging assay and expressed as a mean of EC_50_ values (μg/mL) and respective standard deviation (SD). Statistical analysis was performed by one-way ANOVA followed by Tukey’s test for multiple comparisons. Mean values with the same letters (a, b, c or d) are not statistically different.

Propolis Extracts	G18.EE	G18.EE_70_	G18.HBE	G18.ME	G18.PGE	G18.WE
DPPH^•^ scavenging activity EC_50_ (µg/mL)	10.78 ± 0.43 ^a^	9.91 ± 1.07 ^a^	4.90 ± 0.10 ^c^	1.63 ± 0.27 ^d^	8.17 ± 0.14 ^b^	4.54 ± 0.23 ^c^

EE—ethanol extract; EE_70_—ethanol/water (7:3) extract; HBE—honey brandy extract; ME—mead extract; PGE—propylene glycol extract; WE—water extract.

**Table 5:** Total ortho-diphenols (TOC), phenolics (TPC) and flavonoids (TFC) contents of G18.EE, G18.EE_70_, G18.HBE, G18.ME, G18.PGE and G18.WE. Results are presented as mean ± standard deviation (SD) of mg of gallic acid equivalents per g of extract (mg GAE/g extract) for TOC and TPC, and as mg of quercetin equivalents per g of extract (mg QE/g extract) for TFC. Statistical analysis was performed by one-way ANOVA followed by Tukey’s test for multiple comparisons. For each variable, mean values followed by the same letter (a, b, c, d, e or f) are not statistically different.

Propolis Extracts	Total Ortho-Diphenol Content	Total Phenolics Content	Total Flavonoid Content
	mg GAE/g	mg GAE/g	mg QE/g
G18.EE	263.05 ± 15.19 ^a^	224.60 ± 10.86 ^a^	44.74 ± 1.26 ^a^
G18.EE_70_	367.71 ± 18.86 ^b^	255.30 ± 3.26 ^a^	48.56 ± 0.64 ^b^
G18.HBE	664.16 ± 24.44 ^c^	581.40 ± 35.51 ^b^	50.97 ± 0.26 ^b^
G18.ME	1067.79 ± 37.24 ^d^	1267.57 ± 5.27 ^c^	13.26 ± 0.69 ^c^
G18.PGE	492.30 ± 6.52 ^f^	207.49 ± 8.55 ^a^	34.53 ± 2.43 ^e^
G18.WE	977.71 ± 51.59 ^e^	1261.11 ± 27.86 ^c^	7.40 ± 0.14 ^d^

EE—ethanol extract; EE_70_—ethanol/water (7:3) extract; HBE—honey brandy extract; ME—mead extract; PGE—propylene glycol extract; WE—water extract.

## Data Availability

Data is contained within the article.

## Supplementary Materials

The following supporting information can be downloaded at: https://www.mdpi.com/article/10.3390/molecules27217531/s1, Figure S1: LC-MS chromatographic profiles of the six G18.Es—G18.EE (A), G18.EE70 (B), G18.HBE (C), G18.ME (D), G18.PGE (E) and G18.WE (F)—resulting from the extraction of G18 with different solvents—ethanol extract (EE), ethanol/water (7:3) extract (EE70), honey brandy extract (HBE), mead extract (ME), propylene glycol extract (PGE) and water extract (WE), respectively; Table S1: Chemical composition of G18.EE, G18.EE70, G18.HBE, G18.PGE, G18.ME and G18.WE according to LC-MS analysis.

## Sample Availability

Samples of G18 as well as from the six extracts—G18.EE, G18.EE_70_, G18.HBE, G18.ME, G18.PGE and G18.WE—are available from the authors.
