# Supplementary material for: Propolis Efficacy: The Quest for Eco-Friendly Solvents
Source: Molecules. 2022 Nov 3;27(21):7531. doi: 10.3390/molecules27217531 (PMC9655633; doi:10.3390/molecules27217531)
Supplement: Supplementary file 1 [file molecules-27-07531-s001.zip › molecules-1986576-supplementary.pdf]

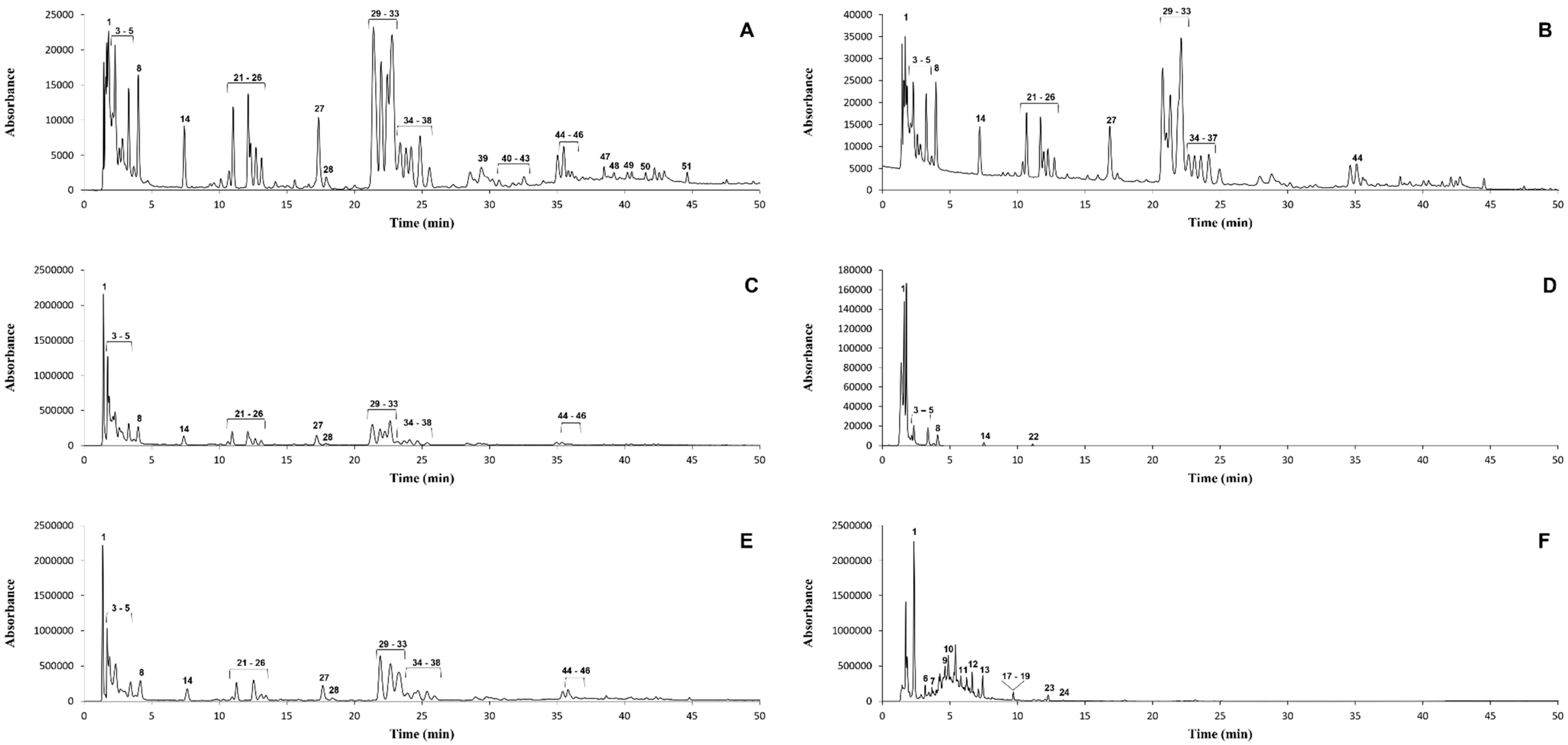

Figure S1. LC-MS chromatographic profiles of the six G18.Es - G18.EE (**A**), G18.EE$_{70}$ (**B**), G18.HBE (**C**), G18.ME (**D**), G18.PGE (**E**) and G18.WE (**F**) - resulting from the extraction of G18 with different solvents - ethanol extract (EE), ethanol/water (7:3) extract (EE$_{70}$), honey brandy extract (HBE), mead extract (ME), propylene glycol extract (PGE) and water extract (WE), respectively.

Table S1. Chemical composition of G18.EE, G18.EE_{70}, G18.HBE, G18.PGE, G18.ME and G18.WE according to LC-MS analysis.

Peaks	t_R (min)	λmax (nm)	$[M - H]^-$ m/z	Main fragments	Compound	Composition (area)					
						EE	EE_{70}	HBE	PGE	ME	WE
**1**	1.9	270	169	125	gallic acid	10912.2	51730.4	3667898.2	3765440.8	453468.7	10924539.4
**2**	2.0	246, 274, 308	481	301, 275	HHDP-hexoside	-	-	-	-	-	2396073.9
**3**	2.7	292, 322	179	135, 163	caffeic acid	74129.4	73335.1	1437945.7	4527696.8	106776.4	1886016.6
**4**	2.9	253, 368	301	257, 229	ellagic acid	20007.4	32798.6	835181.6	477477.1	8809.5	-
**5**	3.1	309	163	119, 145, 108	*p*-coumaric acid	82797	104624.4	1701463.7	2691192.0	133260.7	-
**6**	3.2	275	483	331, 313, 271, 169	digalloyl hexoside	-	-	-	-	-	671609.9
**7**	3.6	271	803	759, 483	gallotannin	-	-	-	-	-	322052.8
**8**	4.1	295, 323	193	177, 149, 133	ferulic acid	110026.5	133297.3	2731698.9	3172370.0	83713.8	-
**9**	4.5	275	707	631, 633	di(HHDP-galloylglucose)-pentose	-	-	-	-	-	144278.0
**10**	4.6	275	551	542, 451, 301	tannin	-	-	-	-	-	474083.0
**11**	5.8	275	551	542, 451, 301	tannin	-	-	-	-	-	700693.9
**12**	6.2	272	433	271, 313, 343, 301, 415	tannin	-	-	-	-	-	357036.2
**13**	6.6	294, 324	469	451, 425, 393, 289	caffeic acid derivative	-	-	-	-	-	1079476.5
**14**	7.3	295, 322	207	163, 148, 133	3,4-dimethyl-caffeic acid (DMCA)	69890.0	71176.4	1614312.6	2345056.4	43166.3	-
**15**	7.3	-	507	331, 313	tannin	-	-	-	-	-	368393.2
**16**	7.4	-	349	163, 145, 119	coumaric acid derivative	-	-	-	-	-	566182.9
**17**	9.7	308	379	193, 175, 134	coumaric acid derivative	-	-	-	-	-	
**18**	9.7	308	273	255, 193, 163	coumaric acid derivative	-	-	-	-	-	607385.2
**19**	9.7	308	439	421, 395, 163	coumaric acid derivative	-	-	-	-	-	
**20**	10.3	288	285	267, 239, 252	pinobanksin-5-methyl-ether	10855.5	-	222098.3	242081.5	-	-

EE - ethanol extract; **EE_{70}** - ethanol/water (7:3) extract; **HBE** - honey brandy extract; **PGE** - propylene glycol extract; **ME** - mead extract; **WE** - water extract; + detected; - not detected or not present.

Table S1. *Cont.*

Peaks	t_R (min)	λmax (nm)	[M - H]⁻ m/z	Main fragments	Compound	Composition (area)					
						EE	EE_{70}	HBE	PGE	ME	WE
**21**	10.7	273, 353	315	300	quercetin-3-methyl ether	22428.5	9572.0	480727.3	431857.4	-	-
**22**	11.0	308	177	-	*p*-coumaric acid methyl ester	91044.5	103837.7	1793831.6	2597572.4	20922.5	-
**23**	12.0	267, 336	269	225, 151	apigenin	81053.6	80167.5	1686629.1	1738039.0	-	552724.4
**24**	12.2	291	271	253, 225	pinobanksin	21581.5	23138.6	676821.1	2081606.2	-	13347.8
**25**	12.5	265, 363	285	285, 257, 151	kaempferol	57741.7	32503.2	843654.2	963450.8	-	-
**26**	13.1	255, 368	315	300	isorhamnetin	41681.6	22732.9	744105.7	631178.9	-	42212.6
**27**	17.1	311	313	298, 269, 257, 241, 179, 153	unknown	93345.2	101377.0	1983529.1	3264842.6	-	11791.8
**28**	18.2	254, 368	329	-	quercetin-dimethyl-ether	32281.7	-	413309.4	521208.5	-	5521.1
**29**	20.9	268, 315	253	209, 181, 225, 151	chrysin	319166.9	356824.0	5047058.3	9783632.2	-	64854.2
**30**	20.9	298, 325	247	179, 135	caffeic acid isoprenyl ester						
**31**	21.9	268, 332	283	268	acacetin	233044.6	194696.0	2056483.6	2318370.9	-	46495.0
**32**	22.4	299, 325	247	179, 135	caffeic acid isoprenyl ester	105843.7	137542.4	908960.2	7817798.0	-	31155.6
**33**	22.9	289	255	213, 211, 151	pinocembrin	320612.3	424858.8	3773026.4	6524866.7	-	197131.4
**34**	23.7	266, 290, 356	269	223, 169, 249	galangin	45020.7	42445.1	525467.1	1005246.9	-	-
**35**	23.9	267, 362	299	284, 165	kaempferide	54817.8	41563.4	649596.2	420278.3	-	1366.4
**36**	24.2	269, 360	329	314, 297, 287, 269, 257	kaempferol-methoxy-methyl ether	69729.3	46438.9	932730.3	814535.0	-	-
**37**	24.9	293	313	253, 271	pinobanksin-3-*O*-acetate	87168.4	54915.5	1136548.7	1977706.0	-	-
**38**	25.6	298, 325	283	179, 135	caffeic acid phenylethyl ester	39397.6	-	688074.0	1049405.2	-	-
**39**	29.5	311	231	163, 119	*p*-coumaric acid isoprenyl ester (isomer)	34049.8	-	339548.3	505212.6	-	-

EE - ethanol extract; **EE_{70}** - ethanol/water (7:3) extract; **HBE** - honey brandy extract; **PGE** - propylene glycol extract; **ME** - mead extract; **WE** - water extract; + detected; - not detected or not present.

Table S1. *Cont.*

Peaks	t_R (min)	λmax (nm)	$[M - H]^-$ m/z	Main fragments	Compound	Composition (area)					
						EE	EE_{70}	HBE	PGE	ME	WE
**40**	30.8	295, 325	295	178, 134, 251, 211	caffeic acid cinnamyl ester	10157.0	-	197881.8	326648.4	-	-
**41**	31.4	268, 305	297<br>403	179, 161, 151, 135<br>-	caffeic acid derivative<br>unknown	4207.4	-	41727.3	83675.4	-	-
**42**	31.9	293	327	253, 271	pinobanksin-3-O-propionate	4215.3	-	75425.8	161658.0	-	-
**43**	32.7	340	269	254, 251, 236, 165	3-hydroxy-5-methoxy flavanone	11217.4	-	165385.1	171407.8	-	-
**44**	35.0	268, 309	387<br>501	281, 267, 255, 293<br>457, 439, 247	unknown<br>unknown	1696.2	4726.6	108929.1	668058.0	-	-
**45**	35.9	290	417	-	methylated pinobanksin-3-*O*-phenylpropionate	6995.1	-	102477.0	272100.6	-	-
**46**	36.4	293	341	253	pinobanksin-3-*O*-butyrate or isobutyrate	4647.6	-	59699.9	255225.1	-	-
**47**	38.6	269, 307, 343	565	283, 269, 281, 417, 455	*p*-coumaric acid-4-hydroxyphenylethyl ester dimer	10609.3	-	70819.0	424620.3	-	-
**48**	39.3	292	355	253, 271	pinobanksin-3-*O*-pentenoate or 2-methylbutyrate	6365.6	-	84997.5	154674.4	-	-
**49**	40.2	297, 320	315	179, 135	caffeic acid derivative	10093.2	-	111209.0	97697.0	-	-
**50**	41.6	280	293	185, 197, 275, 249	*p*-methoxy-cinnamic acid cinnamyl ester	7406.4	-	131351.8	164430.7	-	-
**51**	44.7	310	473	-	*p*-coumaric acid derivative	12766.4	-	38088.3	297478.4	-	-

EE - ethanol extract; **EE_{70}** - ethanol/water (7:3) extract; **HBE** - honey brandy extract; **PGE** - propylene glycol extract; **ME** - mead extract; **WE** - water extract; + detected; - not detected or not pre
